# The Aeroflex: A Bicycle for Mobile Air Quality Measurements

**DOI:** 10.3390/s130100221

**Published:** 2012-12-24

**Authors:** Bart Elen, Jan Peters, Martine Van Poppel, Nico Bleux, Jan Theunis, Matteo Reggente, Arnout Standaert

**Affiliations:** 1 VITO—Flemish Institute for Technological Research, Boeretang 200, 2400 Mol, Belgium; 2 Department of Information Technology (INTEC), Ghent University, Sint-Pietersnieuwstraat 41, 9000 Ghent, Belgium

**Keywords:** measurement bike, mobile sensing, automated data infrastructure, spatio-temporal data, air quality, air pollution, monitoring, mapping, exposure, PM_10_

## Abstract

Fixed air quality stations have limitations when used to assess people's real life exposure to air pollutants. Their spatial coverage is too limited to capture the spatial variability in, e.g., an urban or industrial environment. Complementary mobile air quality measurements can be used as an additional tool to fill this void. In this publication we present the Aeroflex, a bicycle for mobile air quality monitoring. The Aeroflex is equipped with compact air quality measurement devices to monitor ultrafine particle number counts, particulate mass and black carbon concentrations at a high resolution (up to 1 second). Each measurement is automatically linked to its geographical location and time of acquisition using GPS and Internet time. Furthermore, the Aeroflex is equipped with automated data transmission, data pre-processing and data visualization. The Aeroflex is designed with adaptability, reliability and user friendliness in mind. Over the past years, the Aeroflex has been successfully used for high resolution air quality mapping, exposure assessment and hot spot identification.

## 1. Introduction

Because epidemiological studies have consistently demonstrated links between exposure to particulate matter and adverse health effects, ambient air quality limit values have been set and become more stringent. To protect human health, air quality monitoring is regulated by European Directives (2008/50/EC). However, Air Quality monitoring stations are mandatory for a larger agglomeration, whereas spatial variation of air pollutants in a complex urban environment is not covered by one monitoring station. Mobile measurements are applied to complement the stationary air quality measurements at fixed locations, because fixed stations are not capable to depict the full spatial distribution of air pollution over the extent of an urban area for exposure monitoring [1].

The Flemish Institute for Technological Research (VITO) has developed an air quality measurement bicycle called Aeroflex. In this paper we present the Aeroflex. In Section 2 on related work, we cover existing mobile air quality measurement platforms. In Section 3, we describe how we came to the current Aeroflex setup, covering the measurement devices, user interface, and custom logging software. Section 4 deals with the Aeroflex data infrastructure: its data transmission, data storage, automated data pre-processing and automated data visualization functions. Section 5 explains some key challenges of working with both spatially and temporally highly dynamic data, and how they are handled by the Aeroflex. In Section 6 the use of the Aeroflex in a test case in Antwerp to monitor ultrafine particles and black carbon at high resolution is demonstrated. In Section 7 we discuss the fields of application of the Aeroflex. Finally, we end with some conclusions and future plans for the Aeroflex.

## 2. Related Work

Mobile sensing of air pollution is increasingly being applied. Westerdahl *et al.* [2] integrated air pollution measurement instrumentation with a mobile platform to characterize UFP and other pollutants (NO_x_, black carbon) in the Los Angeles (CA, USA) area. Kaur *et al.* [1] quantified the personal exposure of street canyon intersection users to ultrafine particles (UFP), particulate matter (PM_2.5_) and CO in Central London (UK) by mobile data collection by volunteers who travelled a predefined route via five modes of transport at different times of the day, and Zhu *et al.* [3] developed and tested a mobile laboratory to facilitate concurrent measurements of near real time, in-vehicle concentration and size distribution of UFP and other traffic-related air pollutants for health effect studies. Mobile measurements are also applied for high resolution mapping of the spatial variability of air pollution and for the characterization of the local source contributions to the particulate air pollution in urban environments [4–8]. Table 1 gives an overview of mobile air quality measurement platforms which have been used in air quality studies. The used instrumentation, measured parameters and temporal resolution are specified. The Aeroflex, which is described in detail below, differs from the presented mobile air quality platforms because it intends to be a tool that can be broadly used by non-experts with limited air quality monitoring skills, according to their specific requirements. Therefore it combines high adaptability and ease of usage. The Aeroflex also differs from measurement systems designed for community-based sensing.

Most community-based sensing projects (e.g., Common Sense, AIR, OpenSense, CitySense, and EveryAware) designed new measurement devices with low cost sensors for large-scale deployment.

## 3. The Aeroflex

### 3.1. The Aeroflex Concept and Development Phase

The idea behind the Aeroflex is that everybody “able to ride a bike” becomes able to successfully conduct mobile air quality measurements. Since the mobile data collection part is very time consuming, we consider it important that it can be done by anyone. This allows city authorities or companies to have the measurements carried out by their own personnel, in some cases even during their normal daily routine. A certain degree of expertise is still required for device maintenance, resolving of various issues, device re-calibration and data interpretation.

The Aeroflex is developed as a measurement bicycle and not as a measurement car or van such as in [2], [17] and [18]. The reason for this is that the Aeroflex was conceptualized as a mobile platform for local air quality sensing in an urban environment which would be operated in relatively small areas. Furthermore, a bicycle is more flexible, can easily stop at several locations and has access to most public streets. Finally, the bicycle itself is not a source of air pollutants which could potentially interfere with the measurements. The advantages compared to the use of a measurement back-pack, such as in [16], is that longer distances can be covered by bike than on foot, and a higher weight of measurement equipment is possible.

The Aeroflex has been developed and improved in several phases. Mobile air quality measurements have been conducted with Aeroflex measurement bikes by more than ten persons of the VITO staff with varying levels of technical skill both in cities (Antwerp, Ghent, Bruges, Brussels, Louvain-La-Neuve, Tienen, Hasselt, …) [19,20] and in industrial environments (e.g., the Antwerp harbor, stainless steel plant) over a period of five years. It has been used by more than 50 volunteers to carry out rides on predefined tracks (e.g., in the context of the SHAPES project [19,20]). Finally, city personnel of the cities of Ghent and Bruges have used prototypes to map the local air quality. The numerous challenges encountered while conducting those measurements have lead to the current design of the Aeroflex measurement bike.

### 3.2. The Aeroflex and Its Air Quality Sensors

**Figure 1.**
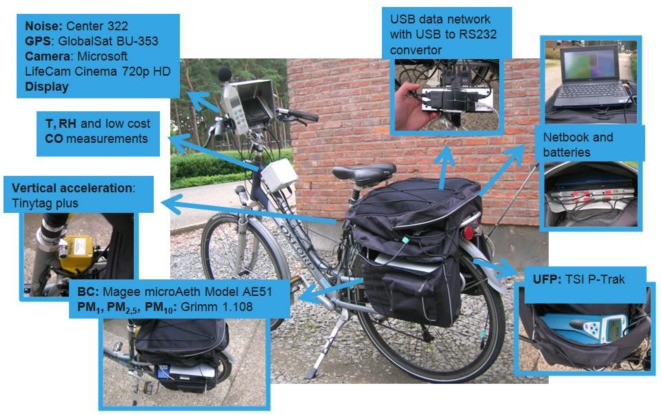
The Aeroflex and its measurement devices.

In Figure 1 the Aeroflex and its measurement devices are shown. The Aeroflex is able to measure a number of air quality parameters:

- Ultrafine particles (UFP) number concentrations with a TSI P-Trak ultrafine particle counter (model number 8525). The P-Track is able to count ultra fine particles in the size range 0.02–1 μm with a time resolution of 1 second. The P-Trak is based on the condensation particle counting technique using isopropyl alcohol [21]. It is a handheld field instrument having a relatively robust performance whilst in motion. It has a rapid warm up, a fast response time, and the ability to detect high concentrations (maximum detectable limit: 500,000 particles cm^−3^). Originally we used to have some problems with the P-Trak. The P-Trak stopped its measurements when being exposed to shocks. The combination of better shock protection and the removal of the P-Trak tilt sensor have resolved this issue. In our experience, the P-Trak is useful for mobile measurement, despite its limited shock resistance [19,20].
- Particulate matter concentrations of different aerodynamic diameter (PM_1_, PM_2,5_, and PM_10_) and the total suspended particulate matter (TSP) with the GRIMM 1.108 dust monitor [22]. The GRIMM 1.108 spectrometer is a portable instrument with two optical sensors which provide near real-time particle number concentration measurements at a maximum measurement rate of 6 seconds. The size range covered by the instrument is 0.23–20 μm over 15 channels. We have successfully used the GRIMM 1.108 on the Aeroflex in studies such as [19,20].
- Black carbon (BC) concentrations with the AethLabs microAeth model AE51 [23]. The microAeth is a pocket-sized black carbon aerosol monitor. The air sample is collected on T60 (Teflon coated glass fiber) filter media, and analyzed in real time. We observed the microAeth is able to conduct measurements with a 1 sec resolution if additional noise reduction techniques are applied during data processing [24].
- CO with an Alphasense CO-BF electrochemical cell. The Alphasense CO-BF CO sensor is a low-cost CO sensor (±60 Euro for the sensor and ±120 Euro for the sensor electronics). We built a measurement device around this sensor called the IDEA sensor box [25]. The CO-BF sensor requires additional temperature correction before it can be used to measure low CO concentrations. We use it on the Aeroflex to experiment with very low cost detection of traffic pollution.

All selected air quality measurement devices are able to transmit their measurements in real time to a computer. Also, their power usage requirements are low enough to power them by batteries. Springs and foam are used to protect the sensitive optical measurement devices from shocks during the measurement ride. Furthermore, the Aeroflex is equipped with a number of additional measurement devices: a GlobalSet BU-353 GPS with SiRFstarIII chipset, a Center 322 sound level meter, a Microsoft LifeCam Cinema 720 p HD camera, a Tinytag Plus high sensitivity vertical acceleration sensor, a temperature, and a relative humidity sensor. Also, a netbook with additional display on the handlebars is used in combination with a USB data network. Both will be discussed in the next sections. Due to the rather large amount of electronic devices used, we choose to use two powerful rechargeable lithium-polymer batteries as a central power supply to power them all. In this way, the Aeroflex has a guaranteed battery autonomy of one working day (8 hours).

### 3.3. The Need for Data Synchronization

Fixed AQ measurement stations typically measure at a time resolution of one hour, 30 minutes; sometimes 1 minute data are available. For mobile measurements a higher temporal resolution (e.g., 1 second) is required, as the distance between subsequent measurements increases when the temporal resolution decreases. Due to this requirement, we want to be sure all measurements are synchronized on a second base. A manual check of all instrument clocks before each measurement ride proved to be too cumbersome and error prone. To resolve this issue, the Aeroflex is able to automatically synchronize all measurement data from all the measurement devices by using a single netbook computer as central data logger. Every second, it reads out all measurement devices simultaneously.

### 3.4. The Need for Adaptability

Due to constant evolution of new measurement devices coming on the market and the specific requirements of each measurement project, it is crucial to be able to easily add, remove and replace measurement devices on the Aeroflex. In the hardware, this was realized by the construction of a USB data network with a tiered-star topology with USB hubs to read out all measurement devices. A USB data network has “plug-and-play” functionality and allows to add a more than 100 measurement devices. The Aeroflex uses a 13 port powered USB hub. More USB hubs can be added to allow the addition of more measurement devices. Where needed, USB to RS232 and USB to RJ45 convertors are added.

**Figure 2.**
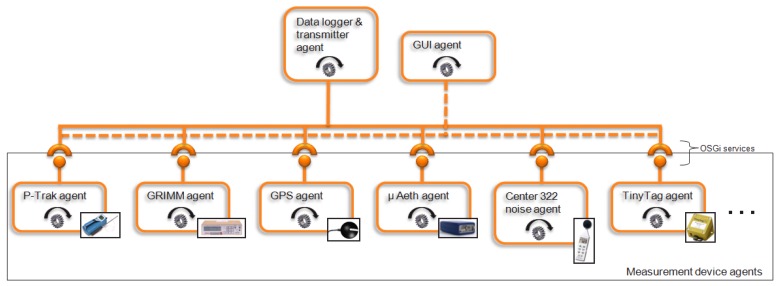
Aeroflex adaptable software architecture with autonomously operating software agents.

On the software side, the Aeroflex software platform has to be able to support an “ever changing” set of measurement devices by detecting new instruments automatically. To meet those requirements, we implemented an autonomous “Measurement device agent” for each measurement device which is responsible for all device specific tasks, including detecting the port(s) on which the measurement device is present, reading out the measurements, and reading out the error codes of the measurement device if available. Each agent is implemented in Java as a separate Open Service Gateway initiative (OSGi) bundle [26] which can be added and removed when needed. Every second, the “Data logger and transmitter agent” pulls the measurements from the “Measurement device agents”. All measurement devices can be read out simultaneously thanks to execution of their “Measurement device agents” in separate execution threads. Also, the “Data logger and transmitter agent” logs the measurements to a data file and/or transmits them over the Internet to the Aeroflex data backend (see Section 3.1). The “GUI agent” is responsible for drawing and updating the graphical user interface. All agents operate completely autonomously in their own thread and are only loosely coupled to each other through OSGi services. An overview of the software platform on the Aeroflex is shown in Figure 2.

### 3.5. Dealing with Failures

In contrast to consumer electronics, air quality measurement devices and their software are often not designed to be very easy to use. This is typically not a big problem since in most cases the technical staff performing the measurements is trained to operate the measurement devices. For the Aeroflex, being easy to use is a key requirement. Everybody who “can ride a bike” should be able to conduct air quality measurements with the Aeroflex. Incorrectly started measurement devices, badly connected cables and device failures during the measurements occurred more frequently than anticipated during the initial test rides. Too often measurement rides had to be redone due to lacking data. Initial experiments with sound alarms to inform the Aeroflex user on failure produced unsatisfying results. Too much noise is present in urban traffic environments to inform the user by sound signals. More success has been obtained by adding a “traffic light” user interface. The display on the handlebars colors green when all measurement devices are operating correctly. It changes color to orange when warnings are present such as “no GPS lock” or minor warnings from measurement devices. And, it changes color to red when measurements should be stopped immediately because of a failing measurement device or cable connection. The concept of a traffic light is quite naturally understood by the persons conducting the mobile measurements: they literally get a “green light” when the Aeroflex is fully operational and mobile measurements can be conducted. Furthermore, the “traffic light” user interface turned out to be easier to observe while biking compared to showing a text message or playing a sound signal.

**Figure 3.**
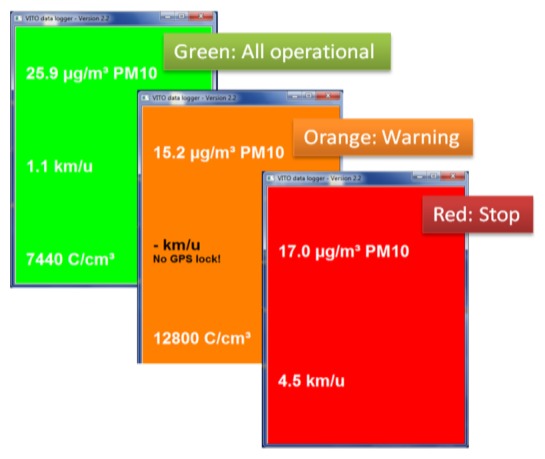
Traffic light GUI.

For each measurement device the current value of at least one measurement value has been added to the traffic light GUI (Figure 3). Although it is quite hard to read measurement values while biking, and although the raw measurements have to be processed and analyzed further for proper interpretation, we noted that the Aeroflex riders are reassured of the correct operation of the used measurement devices when they can see a current measurement value from the devices. Also, by depicting a limited amount of key sensor values, the user can get an impression of the current measurement values.

## 4. The Aeroflex Data Infrastructure

**Figure 4.**
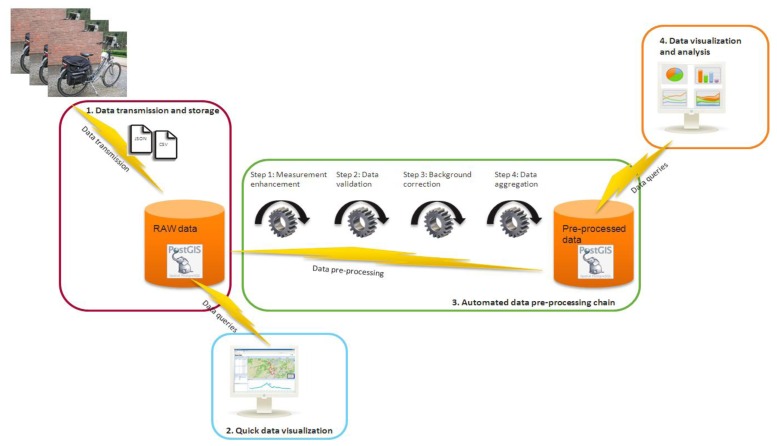
The Aeroflex data infrastructure.

The Aeroflex data infrastructure (Figure 4) contains four parts. First, the data is transmitted to and stored in the data back end. Second, a quick data visualization is offered of the raw data to keep track on measurement progress. Third, the data goes through a data pre-processing chain. Finally, measurement data is visualized and analyzed according to the project requirements.

### 4.1. Data Transmission and Storage

The Aeroflex is able to transmit its measurements in real time and near real time to the data back end. For the real time data transmission, the cellular network is used. The near real time data transmission, at the end of each measurement ride, can also be done over a Wi-Fi network. This allows to follow up the progress of the mobile measurements remotely and to advise the Aeroflex operator from a distance if necessary.

The chosen JSON data format, which is very handy for our automated data back end, was considered as “not enough” by users who wanted to have access to the Aeroflex data for their own analysis. They are used to autonomous measurement devices which generate easy to use data files that can be imported immediately in commonly used data analysis software, such as Excel or R. In addition to the JSON files, all measurements are now also logged on the Aeroflex in CSV format.

### 4.2. Quick Data Visualization

Mobile measurement campaigns may require measurements during a period of weeks or even months. From our experience, the intention to conduct early checks on measurement quality tends to be forgotten due to time constrains and other priorities. The often heard remark is “All data needs to be reanalyzed anyway when the full data set is collected”. Because of this, bad measurement results are often only detected when the measurement campaign has already been finished and can no longer be adjusted.

**Figure 5.**
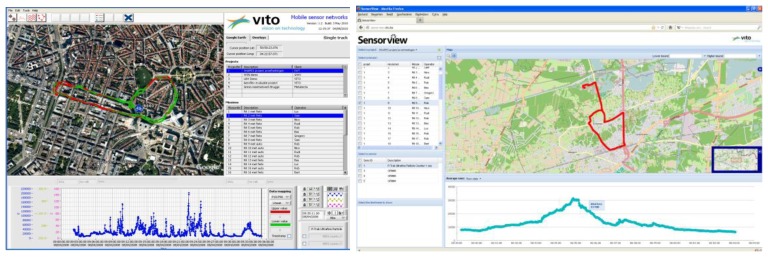
Desktop application for automated visualization of Aeroflex measurements (left). Web application for automated visualization of Aeroflex rides (right).

With the Aeroflex, we solved this issue by making an automated visualization available of the raw measurement data. Each measurement trip is individually visualized on maps and graphs by a LabView based desktop application (Figure 5, left). The Aeroflex measurements can also be visualized on the new VITO SensorView web application, removing the requirement for software installation (Figure 5, right). The applications make use of respectively the OGC Web Feature Service (WFS) and the OGC Web Mapping Service (WMS) to display the measurements. Both web services are offered by a GeoServer instance.

### 4.3. Data Pre-Processing

Before the measurement data is ready for analysis and final visualization, a number of automated data pre-processing steps have to be executed. Currently we have realized a prototype version of an automated data pre-processing chain with four processing steps as illustrated in Figure 4. In the first step, the measurements are enhanced where possible. This includes interpolation and flagging of lacking GPS locations and noise reduction of the microAethalometer black carbon measurements using the ONA algorithm [24]. In the second step, the data is validated by removing incorrect measurements. During the third data pre-processing step, mobile air quality measurements are corrected for available background concentrations. The need for this data pre-processing step is explained in depth in Section 4. In the last step, the measurements are spatially aggregated for each road segment. The results of the data pre-processing chain are stored in a PostGIS database.

### 4.4. Data Visualization and Analysis

**Figure 6.**
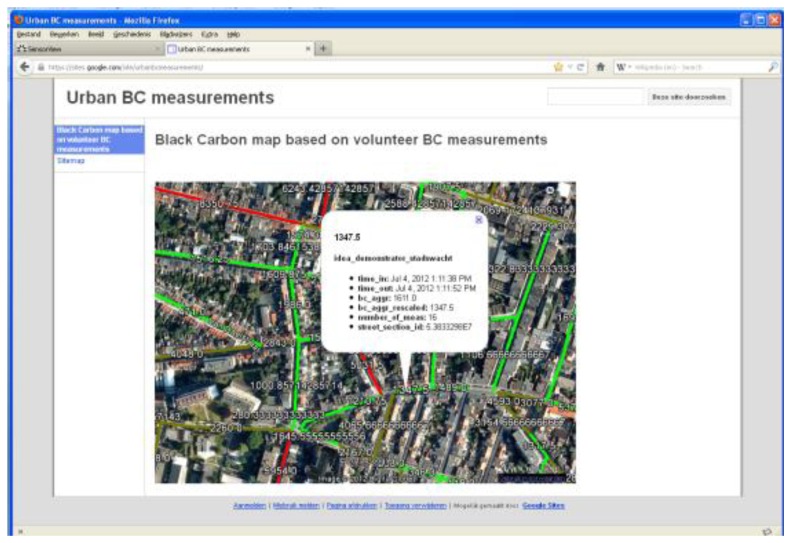
Automated, web-based black carbon map visualization.

The pre-processed data is ready for further analysis and visualization. This step is known to be very project dependent. The research goals of the project determine which analyses and visualizations are required. In Section 5, a limited data analysis is presented of an example of a monitoring campaign using the Aeroflex. For some projects, an automated custom visualization may be required. Figure 6 shows a web visualization of a black carbon map. A BC concentration is assigned to each street based on all mobile BC measurements. Each time new mobile BC measurements are added, the map is automaticaly updated. A GeoServer and the Google Earth plug-in have been used for the automated construction of this webbased BC map.

## 5. Handling Highly Dynamic Spatio-Temporal Data

The measurements of one monitoring run—where a monitoring run is defined as a continuous ride without stops unless forced by traffic conditions—generally provide a spatio-temporal snapshot of the air quality. Given the temporal variability of air pollutants, the timing of the measurement ride has a strong effect on the measured concentrations. For example, a busy street may have lower UFP concentrations than a quiet street, when the former is monitored during night time and the latter during morning rush hours. Moreover, measurements may incidentally be carried out in the wake of a bus or truck. These occasional events may cause a strong peak in the data, which is not necessarily representative for that specific location over a longer period. Two complementary methodologies can be used to reduce the impact of the measurement timing and occasional peak events: the use of (1) an appropriate measurement plan with repeated measurements, and (2) background correction.

An **appropriate measurement plan** with **repeated measurement runs** in the same streets at well set times is required. This way, the spatio-temporal data can be aggregated by means of a summary statistic (median, mean) to obtain a more accurate estimation of the air quality. Obviously, the timing of the measurements is determined by the study objective. To investigate the exposure to air pollutants during commuting hours, measurements should be performed within this time frame on different days. Similarly, the mobile measurements should be distributed over the different hours of a day when the goal is to have a daily mean concentration [27]. Data aggregation results in smoother and probably more accurate maps of the air quality. For the case study presented in Section 5, the measurement runs have been conducted according to a strict measurement plan. When data is collected in a less structured way (e.g., with random measurement runs), it is more challenging to aggregate the collected data in a scientifically sound way.

The **background correction** of the data allows us to compare measurement runs on different days. It involves data rescaling by subtracting a regional background concentration for the observations. As such, the rescaled data quantify the local contribution of traffic and other local sources to the urban air pollution. The variability between monitoring days that is caused by differences in background concentration is therefore reduced. A background concentration is usually obtained from a reference station at a background location, or from mobile measurements made at a background location along the monitoring route [28]. Note that the exact background value is unknown and its estimation contains an error.

## 6. Aeroflex Test Case

In this section we describe a mobile measurement campaign conducted with the Aeroflex. An exploratory analysis of mobile measurements is provided to show some results that can be obtained based on mobile air quality measurements and to assess their potential field of application.

### 6.1. Study Area and Description of the Test Case

A mobile monitoring campaign using the Aeroflex was set up in Antwerp (51°12′N, 4°26′E), Belgium. Antwerp is a medium-sized city (480,000 inhabitants, 985 inhabitants km^−2^). An approximately 2 km long fixed route was defined in a suburb on the Eastern side (Borgerhout) of the city. Borgerhout was chosen because of the presence of a reference air quality monitoring station from the Flemish Environmental Agency (VMM, station 42R801). Preference was given to a short fixed route in the vicinity of a reference monitoring station for comparative reasons. The major part of the mobile route was located in residential area, and streets of differing configuration and with differing traffic dynamics were included in this study (Table 2). The air quality monitoring campaign was performed by 258 runs on 10 days in the period between 2012-02-13 and 2012-03-08. The majority of the runs occurred between 7 am and 1 pm. A total of 248,900 data points were collected.

Two air pollutants were monitored in this study: ultrafine particles (UFP) and black carbon (BC). A TSI P-Trak ultrafine particle counter (model number 8,525) was used to measure the number concentration of UFP within a range from 0 to 5 × 10^5^ particles cm^−3^ at a temporal resolution of 1 second. The P-Trak is based on the condensation particle counting technique using isopropyl alcohol [21]. The flow rate of the P-Trak was 0.7 L minute^−1^, the temperature during operation was within the limits provided by the manufacturer, and alcohol saturation of the wicks was guaranteed. BC measurements were done with a microAethalometer Model AE51 (Magee Scientific) which registers the rate of change in absorption of transmitted light due to continuous collection of aerosol deposit on filter. BC measurements were made at a temporal resolution of 1 second using a flow rate of 150 mL minute^−1^. Filters were changed at the start of each day. A noise reduction algorithm was performed on the BC measurements (ONA algorithm, [24]).

### 6.2. Temporal Analysis of Mobile Measurements

**Figure 7.**
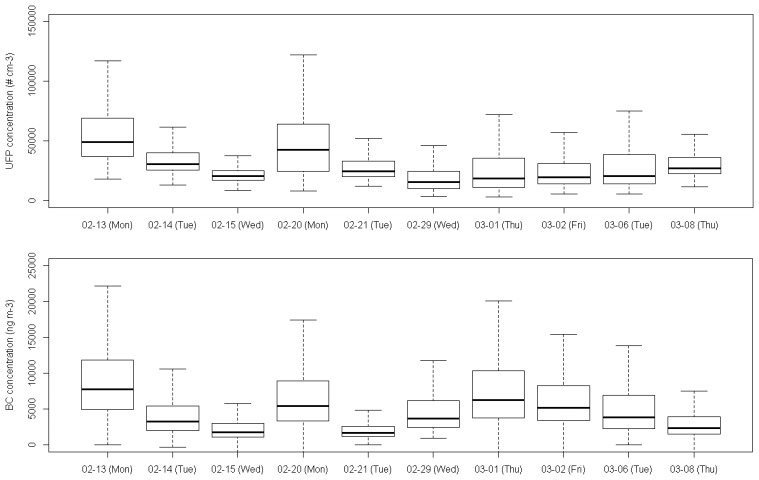
Boxplot of the UFP and BC concentration on the different monitoring days.

The temporal variability in UFP and BC concentrations is present at different levels: between days, between hours of the day and between seconds. Different processes are generally involved in the temporal dynamics of UFP and BC. Local processes related to temporal source dynamics (in urban areas mainly the traffic dynamics), fast transformation processes, e.g., for ultrafine particles or nitrogen oxides, and local atmospheric dispersion cause rapid changes in pollutant concentrations at the local scale, whereas larger scale atmospheric processes determine changes in urban background concentration at a lower spatial and temporal resolution. A comparison of the concentrations grouped by measurement day suggests significant temporal changes (Figure 7). The highest UFP concentrations were measured on February 13th and 20th and the 1st of March, the lowest on February 15th and 20th. The temporal pattern of BC concentrations was similar to that of the UFP concentrations, except for the second BC peak at the beginning of March, which was not observed for UFP. Changes in the urban background account partially for this day-to-day variability. Some of the temporal variability could also be explained by the day of the week. Recently collected traffic count data from a side street of the Plantin en Moretuslei (Provinciestraat, not along the monitoring track) showed increased traffic densities on Mondays.

**Figure 8.**
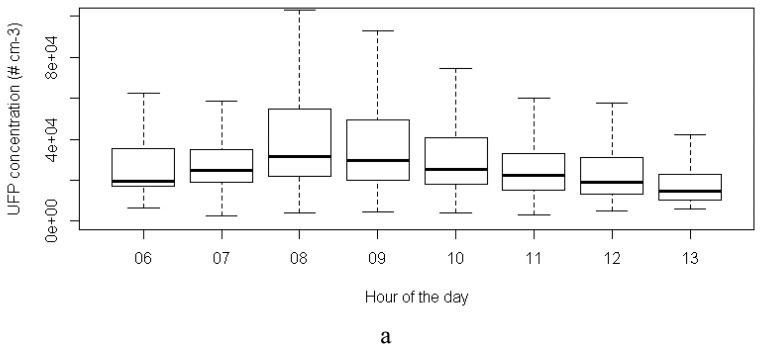

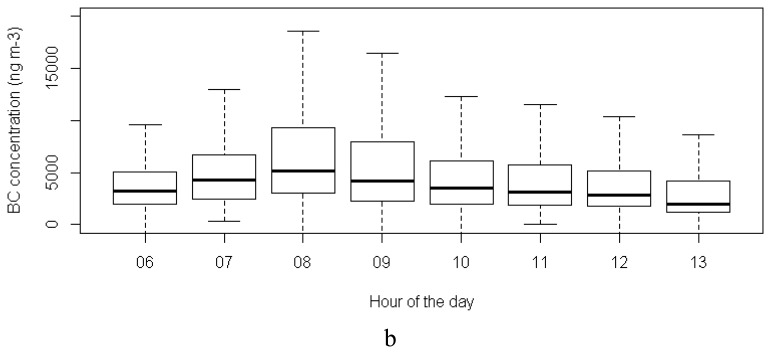
Boxplot of the UFP and BC concentrations in function of hour of the day (merged data from the entire monitoring campaign).

A boxplot analysis of the UFP and BC concentrations in function of the hour of the day reveals the highest concentrations during the morning rush hour (8 am) (Figure 8). The concentrations decrease steadily afterwards. A relatively high variability for all hours of the day (represented by the boxes and whiskers) results from a day-to-day variability between these hours and from short-time fluctuations of the pollutant concentrations within the hour of the day, *i.e.*, short term fluctuations caused by fluctuations in the traffic dynamics. Source emissions in the vicinity of the Aeroflex cause dramatic peaks in the concentrations, but concentrations decline rapidly after these events.

### 6.3. Spatial Analysis of Mobile Measurements

**Figure 9.**
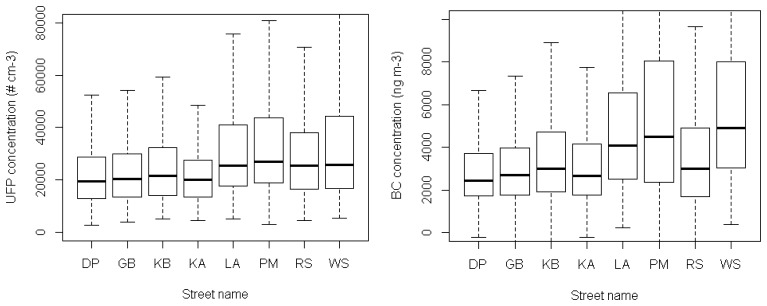

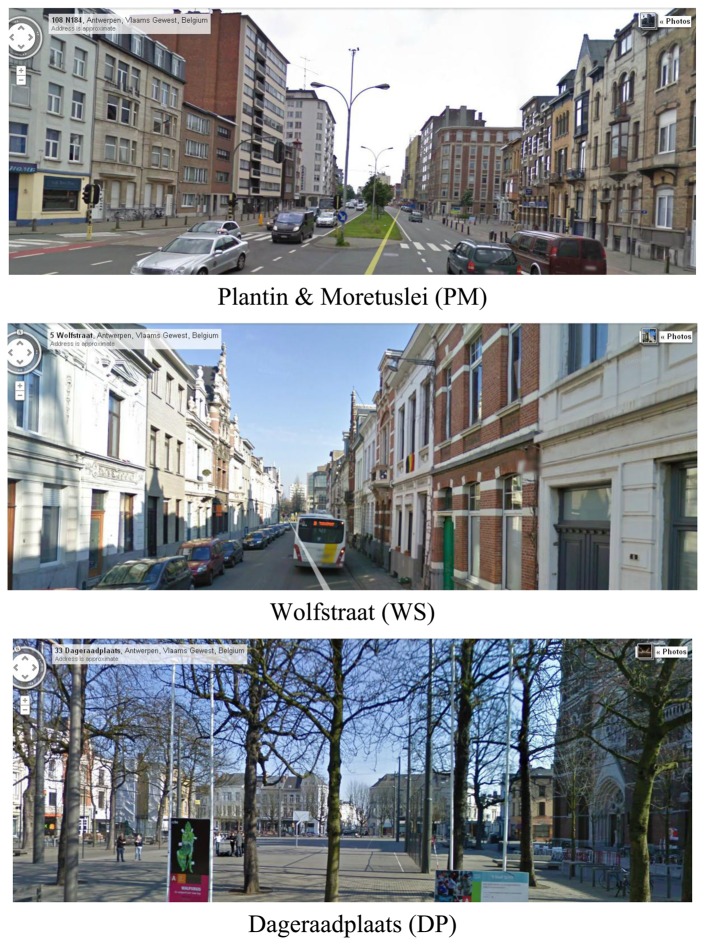
Boxplot of the UFP and BC concentrations for the streets of the monitoring route (merged data from the entire monitoring campaign). DP = Dageraadplaats; GB = Grotebeerstraat; KB = Kleinebeerstraat; KA = Korte Altaarstraat; LA = Lange Altaarstraat; PM = Plantin & Moretuslei; RS = Raafstraat; WS = Wolfstraat.

The mobile measurements have been linked to a street database by their geographical coordinates. As such, the mobile measurements were attributed to the streets of the mobile routes. A streetwise comparison of the UFP and BC concentrations showed that streets with the highest concentrations were Plantin en Moretuslei, Wolfstraat and Lange Altaarstraat (Figure 9). The lowest concentrations were measured at the Dageraadplaats and Korte Altaarstraat. Interestingly, the traffic density differs considerably between Plantin en Moretuslei (43,381 counts per day, see Table 1) and Wolfstraat (5,680 counts per day), yet pollutant concentrations are of a similar magnitude. The distance between source and monitoring platform Aeroflex is much smaller in Wolfstraat where no separate biking lane is present. In addition, Wolfstraat has a more pronounced canyon-like street configuration compared to the wider Plantin en Moretuslei (two lanes in each direction). Therefore, the traffic exhaust at Wolfstraat is measured at higher concentrations and the dilution process is slower. This also illustrates one of the restrictions of the Aeroflex. Its measurements are representative in the first place for the pollutant concentrations that cyclists are exposed to [15].

### 6.4. Comparison with Stationary Measurements

**Figure 10.**
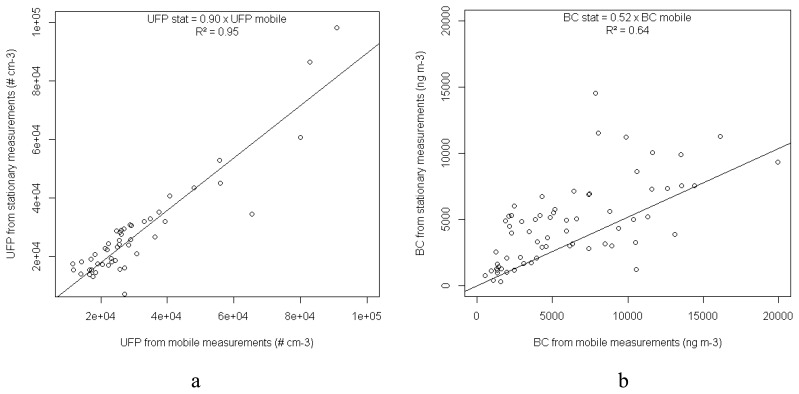
Scatterplots of mobile versus stationary UFP (**a**) and BC (**b**) measurements. Linear regressions are fitted to the data.

Stationary measurements were made concurrently with the mobile monitoring at the VMM reference station at Borgerhout using the same measurement equipment (microAethalometer and P-Trak). The stationary devices were installed on the roof of a monitoring cabin, approximately 3 m high and about 5 m from the traffic. The mobile measurements that were less than 10 m from the permanent station were selected and linked to the stationary measurements based on time. A comparison of these hourly averaged measurements of the mobile and stationary data revealed a reasonably good (BC, R^2^ = 0.64) to a very good (UFP, R^2^ = 0.95) linear correspondence (Figure 10).

### 6.5. Air Quality Map

**Figure 11.**
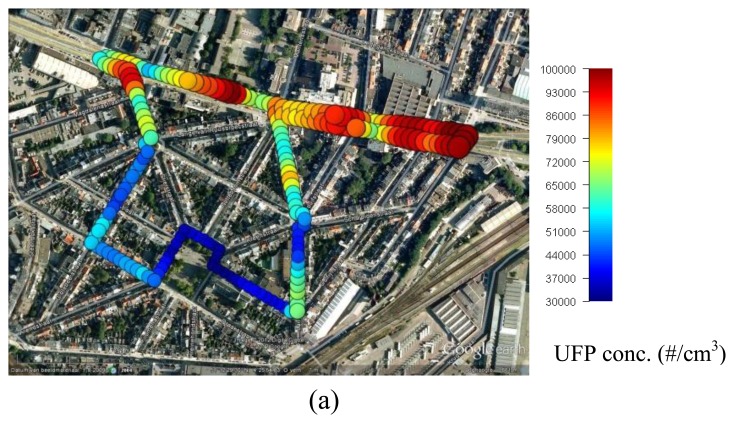

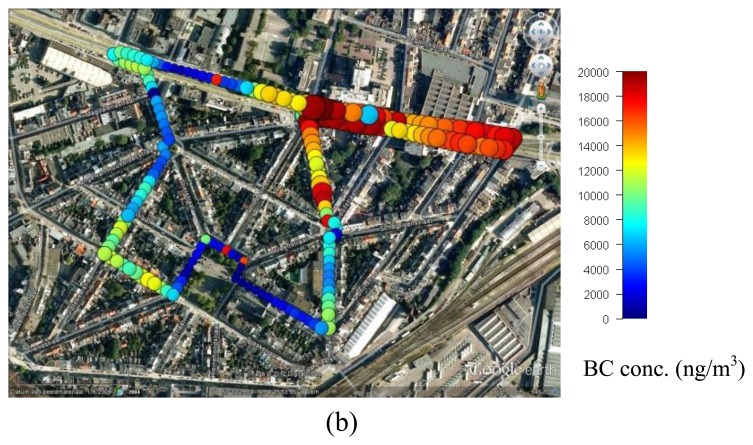
Aggregated maps of mobile UFP (**a**) and BC (**b**) measurements.

Given the high temporal dynamics of air pollutants, a data aggregation step is needed for a clear visualization. Here, a spatio-temporal aggregation of the measurements is performed using a kernel smoothing algorithm to derive daily averaged (*i.e.*, between 7 am and 1 pm) UFP and BC maps for 2012-02-13. A regular lattice was constructed for the monitoring route, consisting of equidistant points with 10 m between the consecutive points. For each of these reference points, a Gaussian kernel with a standard deviation of 10 (arbitrary chosen) was used to weigh the measurements proportionally with their distance to the reference point. As such, the spatial variability of air pollutant concentrations is smoothed to a level which enhances the interpretability (Figure 11). Also, dense measurement clouds due to stopping (for example, for a red light) are aggregated. Plantin en Moretuslei and Wolfstraat had the highest concentrations whereas the lowest concentrations were measured at the Dageraadplaats and Korte Altaarstraat, which is in line with previously described results.

## 7. Applications

Mobile monitoring with the Aeroflex can potentially be used for three main purposes: (1) hot spot identification, (2) high resolution air quality mapping, and (3) exposure monitoring:

### Hot spot identification

Mobile monitoring provides a fast and reliable way to identify and localize emission hot spots. In an urban setting, hot spots may be busy crossroads, industrial plants, *etc.* The high spatial and temporal resolution of mobile monitoring enables to detect these. Also the temporal behavior of hot spots may be monitored by repeated mobile measurements on e.g., different hours of the day, different seasons, *etc.* The Aeroflex is currently used by city authorities to identify and quantify (traffic related) pollution hot spots. This knowledge can be used to improve mobility plans.

### High resolution air quality mapping

The Aeroflex can be used for systematic and high resolution air quality mapping in urban environments. In order to obtain a representative quantification of the air quality in streets, repeated measurements should be conducted (see Section 4). Repetition is needed to allow for data aggregation over a given time frame and spatial extent. Aggregated data (see Figure 12) smooth the eventual occurrence of (abnormal) peak events and thus provide smoother maps that are readily interpretable.

### Exposure monitoring

The Aeroflex has also been used in research projects focusing on personal exposure to air pollutants (such as [19] and [20]). Given the high variability of air pollutants and the determinant effect of the position of the air quality sensors with respect to the pollution sources, Aeroflex data are mostly used to estimate exposure levels of cyclists. Future research will investigate whether these measurements could be rescaled to assess the exposure of pedestrians.

## 8. Conclusions and Outlook

With the Aeroflex, a solution is offered for the limited spatial resolution of fixed air quality measurement networks. The Aeroflex is a mobile air quality measurement platform, allowing measurements with a high spatial and temporal resolution. It is a valuable tool to identify hot spots, map air quality and to monitor exposure. The Aeroflex has successfully been used for mobile measurements in Antwerp, Ghent, Brussels and other cities in Belgium. Analysis of data collected in a test case shows that measurements carried out with the Aeroflex are in line with expected and reported spatio-temporal variability in urban areas.

The Aeroflex contains a number of strong features such as its automated data synchronization, its ability to check for measurement device failures, its strong adaptability and its quite elaborated automated data back end. The most important lessons learned during the development trajectory of the Aeroflex were that both usability, robustness and being able to deal with failures are crucial to obtain good mobile measurements. Although a lot of progress has already been made, we plan to keep these as important points of attention for the future.

For the near future, we see a lot of potential for new application-specific extensions to the Aeroflex data processing infrastructure. Furthermore, we believe that the use of volunteers to conduct mobile air quality measurements during their everyday activities is a promising approach to cover large areas with mobile air quality measurements. Since we have no control over the measurements obtained by volunteers, additional automated data checks and intelligent data processing will be required to obtain high quality results. Further advanced analysis of the collected data will allow to draw conclusions on the number of repeated measurement runs needed to accurately estimate the averaged air quality over longer periods, the potential to distinguish differences in concentrations at street section or sub-street section level, and on the use of background correction methods to increase comparability of measurement results between days.

## Figures and Tables

**Table 1.** Mobile air quality measurement platforms.

**Study**	**Mobile platform**	**Instrumentation**	**Parameters**	**Temporal resolution (s)**
Westerdahl *et al.* (2005, 2007) [2,9]	1998 electric Toyota RAV4 SUV	TSI portable CPC, model 3007, 10	UFP count	10
		TSI CPC, model 3022A	UFP count	10
		TSI Electrical Aerosol Detector, model 3070A	Particle length	2
		Magee Scientific portable aethalometer, model 42	Black carbon	60
		TSI scanning mobility particle system model 3080 classifier:		
		Nano DMA, 3025 CPC 5–153 nm		
		Long DMA, 3025 CPC 16–600 nm	Particle counts	60
		EcoChem PAH analyzer, model PAS 2000	Particulate matter phase PAH	2
		TSI DustTrak	PM_2.5_	10
		Teledyne-API NOx analyzer, model 200e	NO, NOx, NO_2_	20
		TSI Q-Trak Plus monitor, model 8554	CO, CO_2_, temp, humidity	10
Kaur *et al.* (2007) [1]	walking	High-Flow Personal Sampler (HFPS)	PM_2.5_	variable
		TSI P-Trak model 8525	UFP count	1
		Langan (T15 and T15v) CO Measurers	CO, temp	10
Isakov *et al.* (2007) [10]	minivan	Long Pathlength Absorption Spectroscopy-Continuous Flow Analysis system (LPAS-CFA)	Hexavalent chromium	15
		Continuous Flow Analysis (CFA) system	formaldehyde	15
		TSI 3071 Differential Mobility Analyzer and, TSI 3010 Condensation Particle Counter	fine particles (mass)	30
Larson *et al.* [6]	conventional gasoline powered vehicle	particle soot absorption photometer (PSAP) (Radiance Research, Seattle, WA, USA)	Absorption coefficient	1
Hagler *et al.* (2010) [4]	all-electric converted PT Cruiser (Hybrid Technologies, Inc.)	EEPS, model 3090, TSI, Inc.	UFP	1
		Quantum cascade laser, Aerodyne Research, Inc.	CO	1
Dionisio *et al.* (2010) [11]	walking	DustTrak model 8520 monitors (TSI Inc., Shoreview, MN, USA)	PM_10_, PM_2.5_	60
Wallace *et al.* (2009) [12], Adams *et al.* (2012) [13]	van	Thermo scientific model 42i	NO, NOx, NO_2_	10
		Thermo scientific model 48	CO	1
		Monitor labs 8850	SO_2_	1
		GRIMM model 1.107	PM_2.5_	1
Dons *et al.* (2011, 2012) [14,15]	bag on person	microAeth Model AE51, (AethLabs)	BC	300
Vogel *et al.* (2011) [16]	back-pack	GRIMM OPC, GRIMM Nano-Check	UFP, PM_10_, PM_2.5_, PM_1_	60 (UFP) and 6 (PM)
Airparif (2009) [8]	cargo tricycle	Thermo scientific model 42i	NO, NOx, NO_2_	10
		P-Trak	UFP	1

**Table 2.** Description of some street characteristics along the monitoring route.

**Street name**	**Speed limit**	**Configuration**	**Traffic density [day^−1^]** *		

			**light**	**heavy**	**total**
Plantin en Moretuslei	70 km/h	2 lanes, separate biking lane	42,961	420	43,381
Kleinebeerstraat	50 km/h	1 lane	1,269	0	1,269
Lange Altaarstraat	50 km/h	1 lane	5,585	0	5,585
Wolfstraat	30 km/h	1 lane	5,665	15	5,680
Dageraadplaats		Public square, traffic free	NA	NA	NA

* Modelled traffic density averages from the Traffic Centre Flanders.

## References

[1] Kaur S., Nieuwenhuijsen M.J., Colvile R.N. (2007). Fine particulate matter and carbon monoxide exposure concentrations in urban street transport microenvironments. Atmos. Environ..

[2] Westerdahl D., Fruin S., Sax T., Fine P.M., Sioutas C. (2005). Mobile platform measurements of ultrafine particles and associated pollutant concentrations on freeways and residential streets in Los Angeles. Atmos. Environ..

[3] Zhu Y., Hinds W.C., Kim S., Sioutas C. (2002). Concentration and size distribution of ultrafine particles near a major highway. J. Air Waste Manage. Assoc..

[4] Hagler G., Thoma E.D., Baldauf R.W. (2010). High-resolution mobile monitoring of carbon monoxide and ultrafine particle concentrations in a near-road environment. J. Air Waste Manag. Assoc..

[5] Buonocore J.J., Lee H.J., Levy J.I. (2009). The Influence of Traffic on Air Quality in an Urban Neighborhood: A Community University Partnership. Am. J. Public Health.

[6] Larson T., Henderson S.B., Brauer M. (2009). Mobile monitoring of particle light absorption coefficient in an urban area as a basis for land use regression. Environ. Sci. Technol..

[7] Zwack L.M., Paciorek C.J., Spengler J.D., Levy J.I. (2011). Characterizing local traffic contributions to particulate air pollution in street canyons using mobile monitoring techniques. Atmos. Environ..

[8] (2009). Airparif, Influence des aménagements de voirie sur l'exposition des cyclists a la pollution atmospherique.

[9] Westerdahl D., Fruin S.A., Fine P.L., Sioutas C. (2007). The Los Angeles Interantional Airport as a source of ultrafine particles and other pollutants to nearby communities. Atmos. Environ..

[10] Isakov V., Touma J.S., Khlystov A. (2007). A method for assessing air toxics concentrations in urban areas using mobile platform measurements. J. Air Waste Manag. Assoc..

[11] Dionisio K.L., Rooney M.S., Arku R.E., Friedman A.B., Allison F.H., Vallarino J., Agyei-Mensah S., Spengler J.D., Ezzati M. (2010). Within-neighborhood patterns and sources of particle pollution: Mobile monitoring and geographic information system analysis in four communities in Accra, Ghana. Environ. Health Perspect.

[12] Wallace J., Corr D., DeLuca P., Kanaroglou P., McCarry B. (2009). Mobile monitoring of air pollution in cities: The case of Hamilton, Ontario, Canada. J. Environ. Monit..

[13] Adams M.D., DeLuca P.F., Corr D., Kanaroglou P.S. (2012). Mobile air monitoring: Measuring change in air quality in the city of Hamilton, 2005–2010. Soc. Indic. Res..

[14] Dons E., Int Panis L., van Poppel M., Theunis J., Willems H., Rudi T., Wets G. (2011). Impact of time-activity patterns on personal exposure to black carbon. Atmos. Environ..

[15] Dons E., Int Panis L., van Poppel M., Theunis J., Wets G. (2012). Personal exposure to black carbon in transport microenvironments. Atmos. Environ..

[16] Vogel A., Weber K., Fischer C., van Haren G., Pohl T., Schneider F., Pesch M. Innovative optical particle spectrometer to monitor spatial and temporal fine dust and ultra fine particles in low emission zones in Düsseldorf.

[17] Fruin S.A., Winer A.M., Rodes C.E. (2004). Black carbon concentrations in California vehicles and estimation of in-vehicle diesel exhaust particulate matter exposures. Atmos. Environ..

[18] Weijers E.P., Khlystov A.Y., Kos G.P.A., Erisman J.W. (2004). Variability of particulate matter concentrations along roads and motorways determined by a moving measurement unit. Atmos. Environ..

[19] Berghmans P., Bleux N., Int Panis L., Mishra V.K., Torfs R., van Poppel M. (2009). Exposure assessment of a cyclist to PM10 and ultrafine particles. Sci. Total Environ..

[20] Int Panis L., de Geus B., Vandenbulcke G., Willems H., Degraeuwe B., Bleux N., Mishra V., Thomas I., Meeusen R. (2010). Exposure to particulate matter in traffic: A comparison of cyclists and car passengers. Atmos. Environ..

[21] TSI Air Quality: P-Trak Ultrafine Particle Coutern.

[22] GRIMM Aerosol Technique GmbH & Co. KG: GRIMM Portable Aerosol Spectrometer Model 1.108. http://www.grimm-aerosol.com/downloads/datasheets/en/GrimmAerosolTechnik_IAQ_GeneralCatalogue.pdf.

[23] AethLabs MicroAeth Model AE51 Black Carbon Aerosol Monitor. http://www.aethlabs.com/microaeth/.

[24] Hagler G.S.W., Yelverton T.L.B., Vedantham R., Hansen A.D.A., Turner J.R. (2011). Post-processing Method to Reduce Noise while Preserving High Time Resolution in Aethalometer Real-time Black Carbon Data. Aerosol Air Qual. Res..

[25] The Intelligent Distributed Environmental Assessment (IDEA) Flemish IWT project. http://www.idea-project.be/.

[26] (2012). The OSGi alliance. OSGi Core Release 5.

[27] Peters J., Theunis J., van Poppel M., Berghmans P. (2013). Monitoring PM10 and untrafine particles in urban environments using mobile measurements. Aerosol Air Qual. Res..

[28] Van Poppel M., Peters J., Bleux N. (2012). Methodology for mobile air quality measurements in urban environments. Environ. Pollut..

